# Ultrafast Charge Generation Enhancement in Nanoscale Polymer Solar Cells with DIO Additive

**DOI:** 10.3390/nano10112174

**Published:** 2020-10-30

**Authors:** Tongchao Shi, Zeyu Zhang, Xia Guo, Zhengzheng Liu, Chunwei Wang, Sihao Huang, Tingyuan Jia, Chenjing Quan, Qian Xiong, Maojie Zhang, Juan Du, Yuxin Leng

**Affiliations:** 1 State Key Laboratory of High Field Laser Physics and CAS Center for Excellence in Ultra-intense Laser Science, Shanghai Institute of Optics and Fine Mechanics (SIOM), Chinese Academy of Sciences (CAS), Shanghai 201800, China; shitongchao@siom.ac.cn (T.S.); zhangzeyu@siom.ac.cn (Z.Z.); liuzhengzheng@siom.ac.cn (Z.L.); wangchunwei@siom.ac.cn (C.W.); sihaoh@siom.ac.cn (S.H.); jiatingyuan@siom.ac.cn (T.J.); chenjingquan@siom.ac.cn (C.Q.); xiongqian@siom.ac.cn (Q.X.); 2 Center of Materials Science and Optoelectronics Engineering, University of Chinese Academy of Sciences, Beijing 100049, China; 3 Hangzhou Institute for Advanced Study, University of Chinese Academy of Sciences, Hangzhou 310024, China; 4 College of Chemistry, Chemical Engineering and Materials Science, Soochow University, Suzhou, 215123, China; guoxia@suda.edu.cn (X.G.); mjzhang@suda.edu.cn (M.Z.); 5 School of Physical Science and Technology, ShanghaiTech University, Shanghai 200031, China

**Keywords:** nanoscale polymer solar cells, DIO additive, transient absorption spectroscopy

## Abstract

We study the ultrafast photoexcitation dynamics in PBDTTT-C-T (P51, poly(4,8-bis(5-(2-ethylhexyl)-thiophene-2-yl)-benzo[1,2-b:4,5-b′]dithiophene-*alt*-alkylcarbonyl-thieno[3,4-*b*]thiophene)) film (~100 nm thickness) and PBDTTT-C-T:PC_71_BM (P51:PC_71_BM, phenyl-C_71_-butyric-acid-methyl ester) nanostructured blend (∼100 nm thickness) with/without DIO(1,8-diiodooctane) additives with sub-10 fs transient absorption (TA). It is revealed that hot-exciton dissociation and vibrational relaxation could occur in P51 with a lifetime of ~160 fs and was hardly affected by DIO. However, the introduction of DIO in P51 brings a longer lifetime of polaron pairs, which could make a contribution to photocarrier generation. In P51:PC_71_BM nanostructured blends, DIO could promote the Charge Transfer (CT) excitons and free charges generation with a ~5% increasement in ~100 fs. Moreover, the dissociation of CT excitons is faster with DIO, showing a ~5% growth within 1 ps. The promotion of CT excitons and free charge generation by DIO additive is closely related with active layer nanomorphology, accounting for J_sc_ enhancement. These results reveal the effect of DIO on carrier generation and separation, providing an effective route to improve the efficiency of nanoscale polymer solar cells.

## 1. Introduction

The polymer solar cells (PSCs) are attracting great commercial and academic interest due to the clean energy demand and the advantages of light weight, mechanical flexibility and low-cost fabrication [1,2]. The bulk heterojunction (BHJ) nanoscale PSCs using a conjugated polymer as donor and fullerene derivative as acceptor have been demonstrated to realize high power convention efficiency (PCE). Recently, the PCE of single-junction organic solar cells has surpassed 15%, which is due to the significant progress of donor and acceptor materials with complementary absorption bands and high charge-carrier mobility [3]. It has been reported that the nanomorphology optimization of active layer would play a critical role in the performance of the PSCs by improving the charge generation and transport [4,5,6]. Plenty of methods to control the nanomorphology of the active layer have been devised, including post annealing, solvent treatment, interfacial buffer layer and solvent additives. Among them, the use of solvent additives with high boiling points, for example, 1,8-diiodooctane (DIO), is one of the most convenient and simplest methods suitable for large-scale manufacture and compatible with flexible substrates [7,8,9]. DIO has been proved to be an effective method to control not only the bulk nanomorphology but also the surface composition of the BHJ layers [10]. Additionally, a multi-tiered effect of DIO on the blend microstructure and correlated contributions to free carrier formation, transport and recombination has been reported by using photovoltaic characterization, nanomorphology characterization and open circuit corrected charge carrier extraction (OTRACE), suggesting the DIO could affect the photovoltaic performance by a fine-tuning of the blend nanomorphology [8,11,12,13].

Despite the successful investigation of DIO additive on surface composition and photovoltaic properties, the related dynamics influenced by DIO additives about the underlying mechanism for the enhanced performance has rarely been reported. By tracing the behavior difference of charge carriers in the characteristic time scales and for various photo-generated components in complex materials, ultrafast spectroscopy can reveal the individual dynamics and the relationship between different order parameters. For example, it was found that the generation of both interfacial charge transfer states (CTSs) and free polarons in the first tens of femtoseconds and a higher degree of delocalization of the hot CTSs contributing to the probability of charge dissociation within the first 200 fs in PCPDTBT/PC_60_BM [14]. The short lifetime (<1 ps) of delocalized band states was observed in the MDMO-PPV/PC_70_BM, and the driving energy for charge separation in organic photoconversion systems was proved to be the energy needed to reach delocalized band states, which are critical for long-range charge separation [15]. The ultrafast hole transfer (about 3 ps) in the all-polymer blends of J51/N2200 was found to be triggered by a polaron pair-derived hole transfer process, which is different from the EX-dominated channel in the polymer/fullerene systems [16]. These series of works focusing on the ultrafast dynamics in PSCs have shown that the enhanced device performances are closely related to charge dissociation, charge generation rate, and the geminate and bimolecular recombination [16,17,18]. Therefore, in the case of DIO additives, it is also of great importance to reveal the underlying mechanism of DIO on ultrafast charge processes.

In this work, we investigated the ultrafast photoexcitation dynamics influenced by DIO additive in donor PBDTTT-C-T (P51) film and PBDTTT-C-T:PC_71_BM (P51:PC_71_BM) nanostructured blends with a 7.1 fs time-resolved transient absorption measurement. We find that DIO barely affects hot-exciton dissociation and vibrational relaxation in P51 but introduces a longer lifetime for polaron pairs, which are more likely to be dissociated into photocarriers in the blends. Meanwhile, in P51:PC_71_BM nanostructured blends, DIO could increase the CT excitons and free charges generation. Additionally, the dissociation of CT excitons into free charges is also improved by DIO, resulting in J_sc_ enhancement. Our experimental results reveal the underlying mechanism of DIO in effective photocarrier generation, which ultimately leads to higher efficiency of photovoltaic performance. It is expected that the mechanism found in the experiment could provide an alternative route to improve the efficiency of nanoscale polymer solar devices.

## 2. Experimental Section

### 2.1. Material Synthesis and Optical Properties

The polymers were prepared through a Stille coupling reaction between the monomers of 5-alkylthiophene-2-yl-substituted benzo [1,2-*b*:4,5-*b′*] dithiophene (BDT-T) and alkylcarbonyl-substituted thieno-[3,4-b]thiophene (TTC). The donor polymer PBDTTT-C-T (P51) with same weight were dissolved in chlorobenzene, forming the pristine P51 solution. P51 and PC_71_BM were dissolved in chlorobenzene with a 1:1.5 weight ratio and 1,8-diiodooctane (DIO) with a 3% volume ratio was then added to the solutions and stirred before use. The solutions with/without DIO were spin coated on glass substrates with ~100 nm optimal thickness [19,20].

### 2.2. Ultrafast Transient Absorption System

We used a non-collinear optical parametric amplifier (NOPA) to generate visible laser pulses, whose spectral width is broad to support sub-10 fs visible pulses used for the ultrafast transient absorption measurement [21,22]. The laser source of the NOPA system is a commercial regenerative amplifier (Spectra Physics, Santa Clara, CA, USA), whose central wavelength, pulse duration, repetition rate and average output power are 800 nm, 50 fs, 5 kHz, and 950 mW, respectively. A pair of prisms with an apex angle of 68° together with chirp mirrors are used to compress the pulse duration to 7.1 fs. A beam splitter splits the visible laser pulse into pump and probe beams. The intensities of these beams are adjusted to 40 nJ and 4 nJ, respectively. The pump intensity is ~1.27 μJ/cm^2^. Both the pump and probe pulse extend from 538 to 718 nm as shown in Supplementary Materials Figure S1. The probe pulse is dispersed by a polychromator (300 groove/mm, 500 nm blazed) (Princeton Instruments, Trenton, NJ, USA) into a 128-branch fiber bundle, whose other end is connected to avalanche photodiodes (APDs). Therefore, the time-resolved transmittance differences at 128 probe wavelengths are simultaneously detected at the APDs. The signals detected at the APDs are sent to a multichannel lock-in amplifier developed by our group to obtain a signal with a high signal-to-noise ratio. The spectral resolution is ~1.4 nm. The measurement was performed with a probe-delay time from −200 to 2000 fs, and a constant delay time step of 1 fs. All the experiments were performed at room temperature (293 ± 1 K).

## 3. Results and Discussion

**Figure 1:**
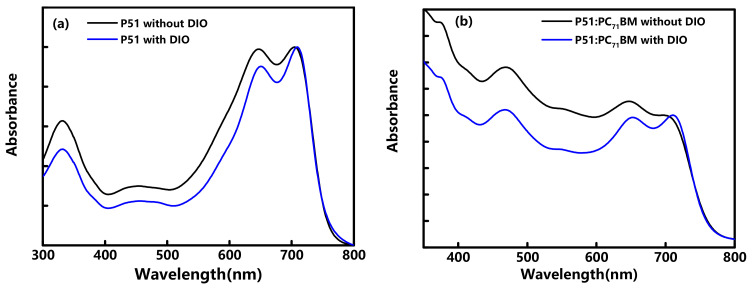
(**a**) Steady-state absorption spectra of the pristine P51 with/without 1,8-diiodooctane (DIO) additive; (**b**) steady-state absorption spectra of P51:PC_71_BM blend with/without DIO additive.

Figure 1 displays the stationary UV-visible absorption spectra of pristine P51 and P51:PC_71_BM blends. As shown in Figure 1a, the P51 exhibits maximum absorbance at ~710 nm, which corresponds to excitation of the P51 singlet exciton. The absorption intensity of P51 film with DIO additives decreases in contrast to the P51 film without DIO additives. For the P51:PC_71_BM blends in Figure 1b, the absorption intensity of the films with DIO additive is decreased in the spectral range from 360 nm to ~710 nm. The absorption spectrum changes could be due to the smaller donor phases with the introduction of DIO, showing that the morphology of P51 and P51:PC_71_BM blends is much more uniform after adding DIO. The shoulder peaks at 710 nm with higher intensity in the P51 and P51:PC_71_BM blends’ films with DIO additive could be attributed from π–π stacking. The spectral range of the laser beam in our transient absorption experiment extends from 538 to 718 nm, indicating both P51 films and P51:PC_71_BM blends could be excited by pump pulses with a photon energy of >1.75 eV (the first absorption gap energy).

**Figure 2:**
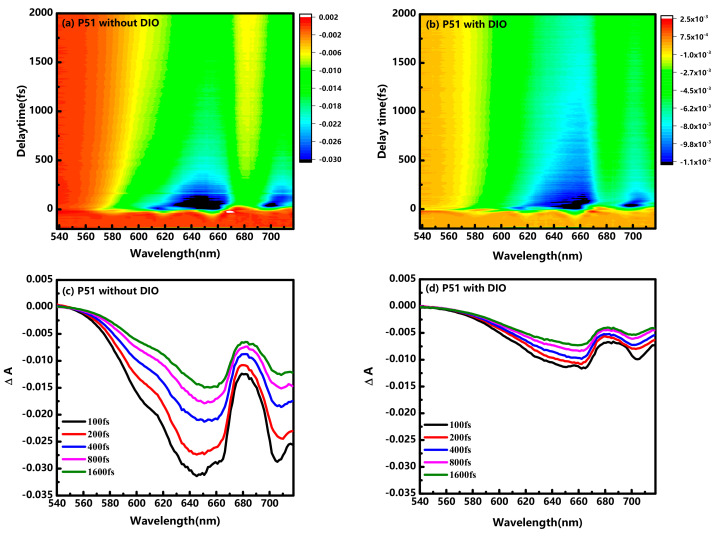
Two-dimensional plot of transient difference absorption ΔΑ (λ, t) and ΔA (λ) spectra at various time delays for (**a**,**c**) pristine P51 without DIO additives and (**b**,**d**) pristine P51 with DIO additives.

**Figure 3:**
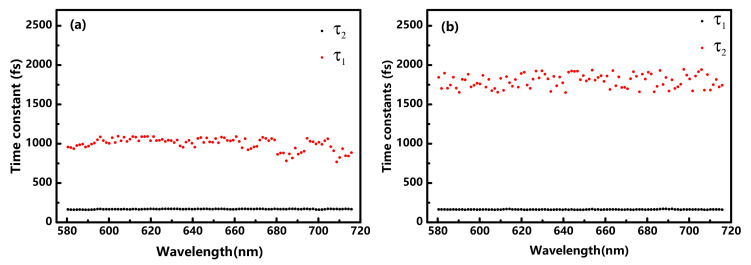
Time constants obtained by a calculation using a two-exponential function for (**a**) P51 without DIO additives; (**b**) P51 with DIO additives.

Figure 2 shows the transient absorption spectra of the donor pristine P51 with/without DIO additives, conducted at ~1.27 μJ/cm^2^ to avoid annihilation effects. The spectra are dominated by two negative peaks, which could be ascribed to ground-state bleach (GSB) and stimulated emission (SE). The GSB has a longer lifetime than SE, because SE occurs only from the excited singlet exciton state; however, the GSB occurs until the ground state fully repopulated. As clearly shown in Figure 2c,d, transient absorption signal in P51 with DIO shows a much weak intensity, nearly one-third of that in P51 without DIO, under the same photoexcitation condition. The decreased signal in Figure 2d could be caused by a reduction of absorption, as shown in Figure 1a or the faster relaxation processes due to the introduction of DIO. To verify the reduction caused by DIO additives on the pristine J51, the transient absorption decay from 65 fs to 2000 fs were fitted with a sum of two exponential functions: $\Delta {A\;=\;A}_{1}\times \exp(-t/\tau_{1})+A_{2}\times \exp(-t/\tau_{2})+B$. Two time constants τ_1_ and τ_2_ obtained from the fitting are shown in Figure 3. The obtained time constants are 162 ± 5 fs, 1000 ± 150 fs for P51 without DIO, respectively. The time constants for P51with DIO are 159 ± 5 fs, 1750 ± 200 fs. At a lower excitation intensity, the rapid decay immediately after the laser excitation can be explained by the hot-exciton dissociation and vibrational relaxation. In the hot-exciton dissociation model [23], the excess photon energy above the singlet exciton is quickly converted into a vibrational heat bath of a polymer segment. Thus, the high effective temperature provides the activation energy to the dissociation of the hot excitons, leading to the formation of polaron pairs within 200 fs [24]. In addition, the excess photo energy for the polaron pair generation is closely related to the exciton binding energy, which is generally accepted to be on the order of 0.5 eV [25,26]. In our experiment, the excess energy is about 0 eV–0.56 eV for photon excitation by the 538–718 nm laser pulse. The excess energy is larger than the exciton binding energy and therefore can cause the formation of polaron pairs from hot-exciton dissociation. Meanwhile, the vibrational relaxation from a higher vibrational state in the lowest singlet exciton state has also been reported within 200 fs [14,24]. In the experiment, the broad excitation covers the absorption peaks of J51 with/without DIO, the remaining hot excitons with excess energy below the threshold of dissociation could decay from a higher vibrational state in the lowest singlet exciton state. Therefore, the ~160 fs rapid decay of the P51 film with/without DIO can be assigned to the relaxation of a higher vibrational state in the lowest singlet exciton state together with hot-exciton dissociation to generate polaron pairs. The rapid processes discussed above in J51 films seems be little influenced by DIO additive.

For the second time constant obtained in the fitting results, we find that the P51 film without DIO exhibits faster decay (~1000 fs) than P51 film with DIO (~1750 fs). The second process could be assigned to the decay of polaron pairs. As discussed above, the hot-exciton dissociation may result in an efficient polaron pair formation. The generated polaron pairs then relax to the ground state via the parallel processes: dissociating into separated polarons, trapping by defect states, and recombination [27,28]. The time constant of defect trapping has been reported to be 2.8 ps in the conjugated polymer P3HT. Additionally, a 950 fs relaxation is reported to be caused by the recombination and dissociating into separated polarons [28]. The polaron pairs cannot be efficiently dissociated into polarons without an applied electric field because of the strong Coulomb attraction. Hence, in our experiment, the decay of the polaron pairs within 2000 fs is consisted of the recombination process (~950 fs) and trapping by defect states (~2.8 ps). The longer second time constant in P51 film with DIO implies that the polaron pairs have a longer lifetime rather than recombine to the ground state, which are more likely to be dissociated into photocarriers as will be discussed in the blends. Therefore, the distinct decrease in transient absorption signal in the pristine P51 film with DIO could be assigned to the reduction of stationary absorption because the relaxation processes with DIO has been elucidated to be slower than the P51 without DIO. Moreover, the introduction of DIO in P51 brings a longer lifetime of polaron pairs, which could contribute to photocarrier generation.

**Figure 4:**
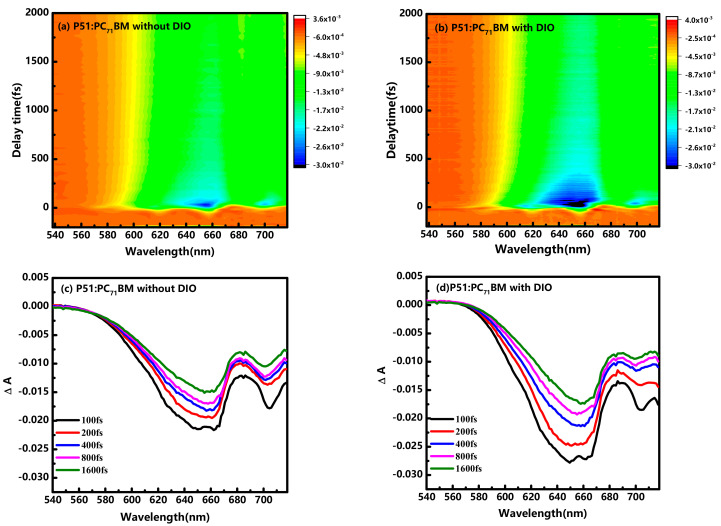
Two-dimensional plot of transient difference absorption ΔΑ(λ, t) and ΔA(λ) spectra at various time delays for (**a**,**c**) P51:PC_71_BM without DIO additives and (**b**,**d**) P51:PC_71_BM with DIO additives.

Turning now to the influence of DIO on the P51:PC_71_BM blends, the transient spectra were quite different after adding DIO in the P51:PC_71_BM blend. In the previous work, the enhanced device performance after adding DIO additive has been reported be related to the bulk nanomorphology and surface composition of the blend film [10]. Moreover, the addition of DIO has been demonstrated to lower energetic disorder in the P51:PC_71_BM blend, which can suppress the recombination of triplet charge-transfer states, and hence enhance device performance [29,30]. In order to reveal the underlying mechanism of DIO on the P51:PC_71_BM blend, the transient absorption spectra of the P51:PC_71_BM blend with/without DIO additive was first shown in Figure 4. Figure 4a,b displays the time (−200 to 2000 fs) and wavelength (538 to 718 nm) resolved spectra of P51:PC_71_BM blends with/without DIO additive, respectively. At the same lower excitation intensities of ~1.27 μJ/cm^2^, the blends with/without DIO additives exhibit two negative peaks at ~650 and ~705 nm, which is similar with the results observed in pristine P51, indicating that polymer P51 plays a major role in the photoexcitation within 2000 fs. The negative peaks could be assigned to GSB and SE due to the overlap of the stationary absorption spectra. As clearly shown in Figure 4c,d, the blend with DIO that exhibits an increased signal though the absorption intensity is weaker than the blend without DIO, which suggests that the faster relaxation processes are in GSB and SE. Moreover, in contrast to pristine P51 shown in Figure 2c,d, the ratio of the second negative peak intensity (~705 nm) is weaker in the blend than that observed in pristine P51, which could be ascribed to the reduction of SE feature as excitons splitting could occur in the blends on the ultrafast timescale. Especially, the blend with DIO shows a smaller ratio of negative peak at ~705 nm than that without DIO, as depicted in Figure 4c,d, indicating that a more efficient exciton splitting could exist in the blend with DIO.

**Figure 5:**
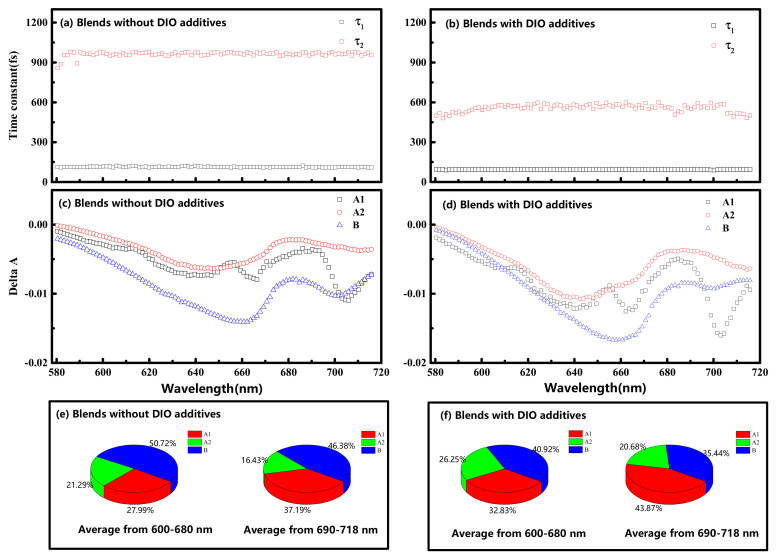
Fitting results with the two-exponential function for the time traces: (**a**) P51:PC_71_BM blends without DIO additives; (**b**) P51:PC_71_BM blends with DIO additives; (**c**) decomposed Α1, A2 and B in (**c**) blends without DIO additives (**d**) blends with DIO additives. (**e**) Percent of Α1, A2 and B in blends without DIO additives; (**f**) percent of Α1, A2 and B in blends with DIO additives.

To quantify the effects of DIO additives on the blends, a sum of two exponential functions: $\Delta A(t){\;=\;A}_{1}\times \exp(-t/\tau_{1})+A_{2}\times \exp(-t/\tau_{2})+B$ was also used to extract the precise time constant within 2000 fs, where A1, A2 and B correspond to the decomposed amplitude of the first decay process, amplitude of the second decay process and constant amplitude, respectively. As shown in Figure 5a,b, the time constants in the P51:PC_71_BM blend without DIO are 113 ± 5 fs, 943 ± 60 fs, respectively, while the blend with DIO shows two faster decay processes with time constants 93 ± 8 fs, 533 ± 50 fs. The delay time dependence of the normalized difference absorbance for the P51:PC_71_BM blend with/without DIO additives was recorded at four different wavelengths, as shown in Figure S2. The time traces contain two exponential decay reflecting carrier relaxation dynamics and oscillations reflecting vibration dynamics, which is often observed by using the excitation and probe pulse with pulse duration < 10 fs [22,31,32]. All the time-resolved signals with DIO additives demonstrate a faster decay process, suggesting that the introducing of DIO additives could accelerate the carrier relaxation.

The first decay ~100 fs is shorter than that in pristine P51 because efficient charge carriers could be generated on <100 fs time scales in the blend [14]. The well-accepted model system of polymer/fullerene blends has been described within a modified Marcus framework, following four elementary steps: (i) photon absorption, creating a spatially localized Frenkel exciton in the donor phase; (ii) exciton diffusion to the donor–acceptor interface; (iii) exciton dissociation at the interface leading to the formation of an interfacial charge-transfer states (CTSs), leaving an electron in the fullerene region and a hole in the polymer region; and (iv) dissociation of these CTSs into free charges [16,33]. However, it has also been recognized that ultrafast long-range charge separation through the role of delocalization of wave functions [34,35]. The ultrafast yield of free charges has been reported to be weakly dependent on the energetic driving force but strongly dependent on fullerene aggregate size and packing [35]. In our experiment conducted at ~1.27 μJ/cm^2^, the first decay process could be assigned to the CT excitons generation and free charges generation from the hot exciton decay. It has been reported that singlet excitons containing significant CT configurations are known as CT excitons, which are considered to play an import role in photocurrent generation of charge carriers [24]. In the blends, due to the large interchain interaction caused by dense π stacking in crystalline domains, the CT excitons are more likely to be formed at the donor–acceptor interface within 100 fs. Meanwhile, delocalization on fullerenes is predicted to have a significant impact on charge separation within 100 fs, which is sensitive to fullerene cluster size and blend nanomorphology [33,36]. Moreover, as shown in Figure 5c,d, the decomposed A1 dependent on wavelength also exhibits a higher peak at ~705 nm, which is closely to the SE signal. The first decay from 93 to 113 fs after adding DIO is consistent with the assignment of free charge generation because the blend film nanomorphology processed with DIO has shown higher surface roughness in our previous work [10]. In addition, the modification of film nanomorphology by DIO is also likely to cause the larger CT configurations of singlet excitons in the donor P51. Hence, the first decay of the two blends is assignable to CT exciton generation and free charges generation in SE, and the effect of DIO on the blend may accelerate the process.

Subsequently, as shown in Figure 5a,b, the obtained second lifetime varies from 943 to 543 fs after the introduction of DIO. The lifetime within 1 ps is quite different from that in the pristine J51, which could be ascribed to the dissociation of CT excitons into free charge carriers. Theoretically the CT states could either recombine to the ground state, a process termed geminate recombination, or dissociate into free charge carriers that can be extracted from the blend as photocurrent. The geminate recombination is much smaller in the blends than dissociation within 1 ps as the CT emission can be quenched with a minor photoluminescence (PL) peak at 720 nm in the P51:PC_71_BM blends [30,37]. In addition, as shown in Figure 5c,d, the spectral shape of the decomposed A2 shows a similar shape of A1 in the range from 580 to 700 nm, which is consistent with the CT excitons in ~100 fs. In previous work, the dissociation of CT excitons within 1 ps has been observed in many fullerene-based blends [14,35,38]. In the similar PBDTTT-C/PC_60_BM and PBDTTT-CT:PCBM blends system, the time of exciton dissociation within 1 ps is also reported by a combination of fs-μs broadband vis-NIR transient absorption (TA) pump−probe spectroscopy and multivariate curve resolution (MCR) data analysis [39,40]. For the constant B, it represents a long-life-time component—being beyond the range of our time measurement, which could be attributed to the recombination decay of photocarriers in the blends.

According to the discussion above, the introduction of DIO accelerates the dissociation of CT excitons within 1 ps. Besides, as shown in Figure 5e,f, a nearly 5% increase in the CT exciton generation and free charge generation in the first ~100 fs occurs, as does a nearly 5% increase in the dissociation of CT excitons in the blend with DIO additives, corresponding to the 10% decrease in the decay of photocarriers. This implies that, with the assistant of the DIO additives, primary hot singlet excitons generated in blends are more likely to be dissociated into photocarriers that further produce a photocurrent, which is consistent with the increase in J_sc_ value as reported in density-voltage (J–V) measurement [10,30]. The enhanced ultrafast photocarrier generation influenced by the DIO are closely related with the increased domain size of donor/acceptor components, lower levels of energetic disorder, and optimized surface compositions in previous work [10,19,30]. Considering the decreased stationary absorption of P51:PC_71_BM blends with DIO, it is reasonable to conclude that even though the reduction of the absorbed photons, the effect of the DIO additive on the P51:PC_71_BM blend demonstrates an increasement of photocarrier generation.

**Figure 6:**
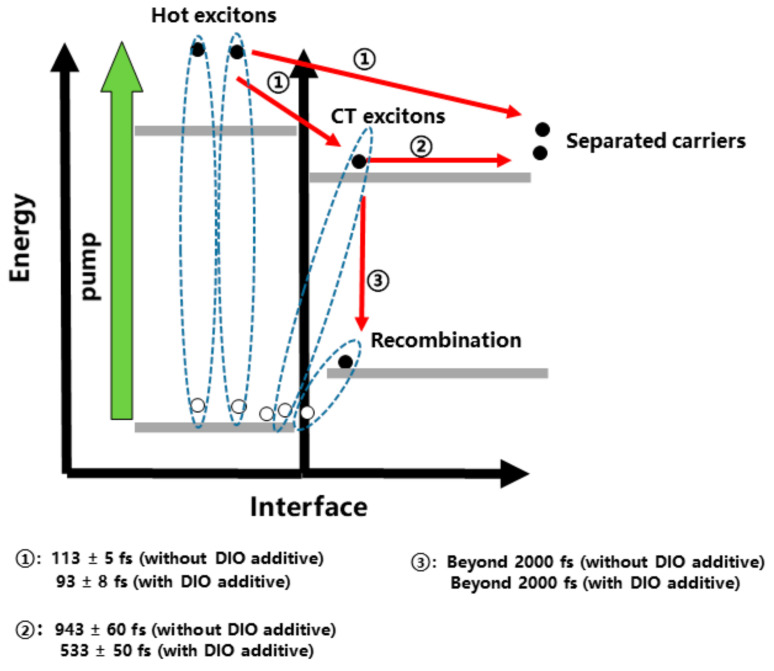
A schematic of ultrafast interfacial excitons dynamics in the P51:PC_71_BM blend after photoexcitation. ①: Charge Transfer (CT) excitons and free charge generation from hot excitons; ②: dissociation of CT excitons into free charge carriers; ③: recombination decay of photocarriers.

Based on above discussion, we can rationalize the charge generation process at the very initial timescale on photoexcitation. As illustrated in Figure 6, hot singlet excitons are generated after above-bandgap photon absorption, which then decay with the generation of CT excitons and free charges with a lifetime of ~100 fs. The introduction of DIO could accelerate the generation time from 113 fs to 93 fs with an increasement of ~5% of the decomposed A1. The dissociation of CT excitons is observed within 1 ps. DIO could contribute to the dissociation with a decreased time from 943 fs to 533 fs and an increasement of ~5% of the decomposed A2. The longer lifetime beyond our time measurement could be mainly ascribed to recombination decay of photocarriers. Meanwhile, the percent of the B is decreased by 10% after introducing DIO.

## 4. Conclusions

In summary, we used sub-10 fs time-resolved spectroscopy to investigate the effect of DIO additives on the P51 and P51:PC_71_BM nanostructured blends. Although DIO barely affects the hot-exciton dissociation into polaron pairs and vibrational relaxation at ~160 fs scale, the longer lifetime (1750 fs) of polaron pairs is shown in P51 with DIO, which could promote to the photocarrier generation rather than recombination. In the P51:PC_71_BM nanostructured blends, DIO demonstrates a beneficial effect on CT excitons and free carrier generation within 100 fs together with a ~5% enhancement. The lifetime of CT exciton dissociation decreases from 943 to 533 fs and a ~5% increasement is observed in the blends with DIO. The carrier generation after photoexcitation within 1 ps is affected by DIO, which could be closely related to the increased domain size of donor/acceptor components. Lower levels of energetic disorder were found in our previous work. The results reported in this work may suggest the key role of additives for ultrafast charge generation and dissociation in the active layers and pave a route to efficient nanoscale polymer solar cells.

## Supplementary Materials

The following are available online at https://www.mdpi.com/2079-4991/10/11/2174/s1, Figure S1: Laser spectrum of the probe beam used in the transient absorption measurement. Figure S2: Normalized of the blend without DIO additives (black line), the blend with DIO additives (blue line) and the fitting results (red line) probed at four different wavelengths.
