# Supplementary material for: Ultrafast Charge Generation Enhancement in Nanoscale Polymer Solar Cells with DIO Additive
Source: Nanomaterials (Basel). 2020 Oct 30;10(11):2174. doi: 10.3390/nano10112174 (PMC7692121; doi:10.3390/nano10112174)
Supplement: Supplementary file 1 [file nanomaterials-10-02174-s001.pdf]

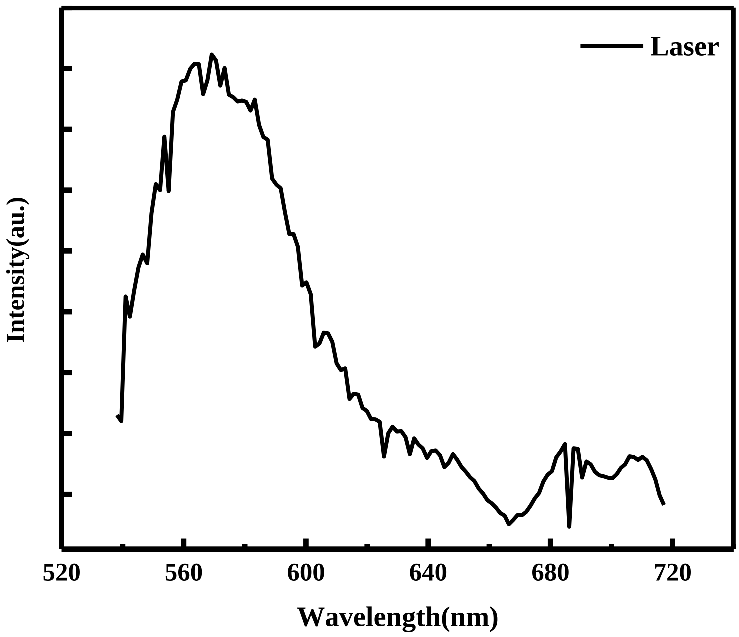

Figure S1. Laser spectrum of the probe beam used in the transient absorption measurement.

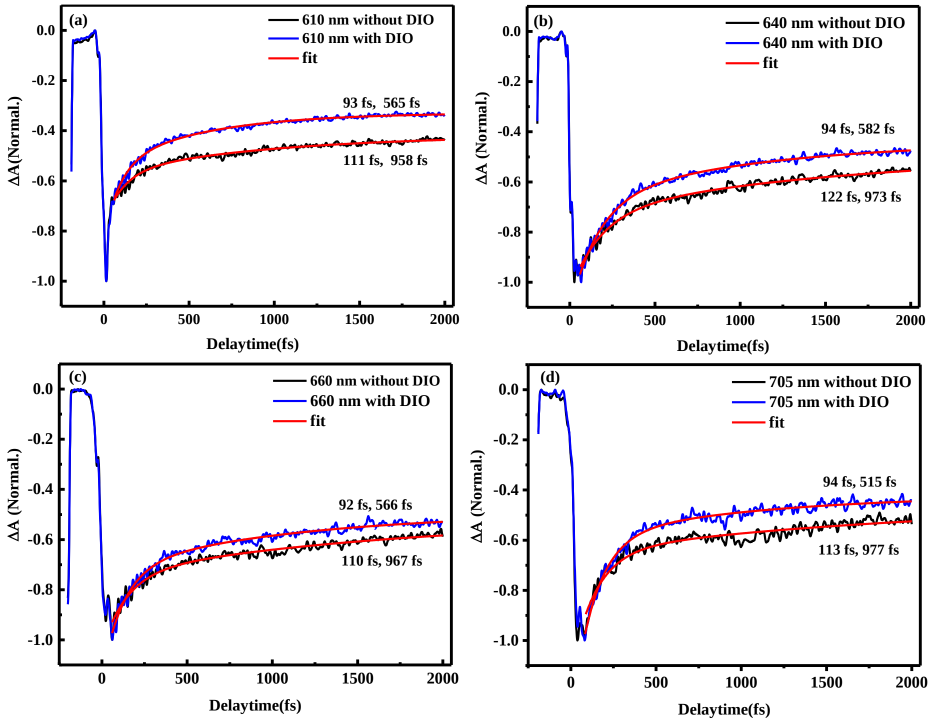

Figure S2. Normalized of the blend without DIO additives (black line), the blend with DIO additives (blue line) and the fitting results (red line) probed at four different wavelengths.
